# The application of robot-assisted microsurgery in orthoplastic surgery

**DOI:** 10.1515/iss-2025-0030

**Published:** 2026-01-20

**Authors:** Felix Struebing, Benjamin Thomas, Emre Gazyakan, Florian Falkner, Simon Mayer, Charlotte Holup, Arne Boecker, Ulrich Kneser

**Affiliations:** BG Klinik Ludwigshafen, Department of Hand, Plastic, and Reconstructive Surgery, Burn Center at Heidelberg University, Ludwigshafen, Germany; Heidelberg University, Heidelberg, Germany

**Keywords:** robotic microsurgery, robot-assisted microsrguery, reconstructive microsurgery, orthoplastic surgery, microvascular reconstruction

## Abstract

**Objectives:**

The orthoplastic approach has emerged as the gold standard in the reconstruction of complex extremity injuries. While robot-assisted microsurgery (RAMS) enhances precision and ergonomics, its role in the challenging orthoplastic setting remains largely unexplored. This study aimed to evaluate our initial institutional experience with robot-assisted free flap reconstruction for complex limb injuries within an orthoplastic approach.

**Methods:**

From February 2023 to February 2025, we prospectively collected data on patients with combined traumatic bone and soft tissue injuries undergoing robot-assisted free flap transfer to the extremities. Data included patient demographics, surgical details (e.g., operating times, anastomosis/coaptation duration), and complications. Ethical approval was obtained.

**Results:**

Our cohort of 18 patients (78 % male, mean age 50.3 years) received free flaps for traumatic soft tissue defects (78 % lower extremity). The ALT flap was most commonly used (67 %). Mean operating time was 353.0 min. 19 anastomoses and coaptations were performed, with no conversions to conventional techniques. A major complication (arterial thrombosis) occurred in one case, successfully salvaged with conventional re-anastomosis, resulting in no complete flap losses. There was one partial flap loss. Three minor complications (delayed wound healing, 17 %) were observed.

**Conclusions:**

Our experience shows the feasibility of RAMS in orthoplastic limb reconstruction. Further studies are needed to validate these findings and define the optimal role and benefits of RAMS in orthoplastic surgery.

## Introduction

Orthoplastic surgery is a specialized, multidisciplinary field that combines the principles and techniques of orthopedic and plastic surgery to improve the management of complex limb injuries. This integrated approach emphasizes close collaboration between orthopedic and plastic surgeons to achieve superior functional and aesthetic outcomes while minimizing the risk of complications [1]. First formally conceptualized by Levin in 1992, the orthoplastic model has since evolved into a standard of care in many level 1trauma centers with significant reconstructive case load, particularly for cases involving severe trauma, infection, or non-union, where limb salvage can be particularly challenging and the risk of amputation remains high [2], 3].

By jointly addressing skeletal stability and soft tissue coverage, the orthoplastic paradigm has demonstrated clinical benefits such as reduced infection rates, shortened hospital stays, decreased time to union, and improved quality of life [1], 4], 5]. These outcomes are especially critical in the context of limb-threatening injuries where timely and coordinated intervention can determine the success of limb preservation efforts.

In parallel with the evolution of surgical strategies, technological innovation has expanded the frontiers of microsurgical practice. One of the most notable advancements in the last yearsis the emergence of robot-assisted microsurgery (RAMS). This technology offers unique advantages, including tremor elimination, motion scaling, and improved ergonomic conditions, which collectively enhance surgical precision and reduce surgeon fatigue. The Symani Surgical System (Medical Microinstruments, Inc., Wilmington, DE, USA) and the MUSA system (MicroSure, Eindhoven, Netherlands) currently are the only clinically utilized robotic platforms specifically developed for microsurgical applications. These systems have been utilized in a broad range of clinical procedures, including microvascular soft tissue reconstruction [6], [7], [8], [9], [10], [11], peripheral nerve surgery [12], [13], [14], breast reconstruction [15], 16], and lymphatic surgery [17], [18], [19].

Despite this progress, the application of robot-assisted microsurgery within the orthoplastic framework remains underexplored. While RAMS has been studied in preclinical models and select clinical settings – such as breast reconstruction, lymphatic surgery, and head and neck reconstruction – its role in complex limb reconstruction, particularly in trauma and post-infectious scenarios, has not been systematically assessed. The orthoplastic setting presents unique challenges, including variable recipient anatomy, contaminated fields, and the need for intraoperative adaptability – factors that may either benefit from or challenge robotic implementation.

To address this gap, we conducted a retrospective analysis of extremity reconstructions performed at our institution using the Symani Surgical System in conjunction with an orthoplastic surgical approach. Our objective was to evaluate the implementation of robot-assisted free flap reconstruction in the cases of complex limb injuries. By providing the first institutional experience of RAMS in orthoplastic surgery, this study aims to establish a foundational framework for its clinical integration and to inform future investigations into its potential role in limb salvage strategies.

## Materials and methods

### Data collection

A prospective database was established to document and facilitate access to all sequential cases of robot-assisted microsurgery performed at our institution from February 2023 to February 2025. The analysis included all patients with combined soft tissue and bone injuries who required osteosynthesis and subsequently underwent soft tissue reconstruction via free flap transfer to the extremities using the Symani Surgical System. Patients with soft tissue defects resulting from infection or osteomyelitis were excluded from the study. Data on patient demographics (age, sex, body mass index (BMI), comorbidities, risk factors) and surgical details (type of surgery, type of microsurgical reconstruction, operating times, duration of anastomosis or coaptation, number of stitches, surgical outcomes including intra- and postoperative complications) were collected. The study protocol complies with the Declaration of Helsinki, and Ethical Approval was secured from the local ethics committee (Medical Commission Rhineland-Palatinate, Mainz, Germany; Approval number: 2023–16997).

### Perioperative protocol

The Symani Surgical System was utilized in all cases. The microsurgical reconstructions were carried out using standard flap raising techniques as previously described. The standard microinstruments of the Symani Surgical System were used in all cases, super-microsurgical instruments were not used in this study. Optical magnification was obtained either through conventional microscopy (Mitaka MM51, Mitaka Kohki Ltd., Tokyo, Japan) or digital exoscopy using 4K-3D screens (ORBEYE, Olympus, Tokyo, Japan).

### Statistical analysis

Continuous variables are presented as means with standard deviation (SD) or medians with interquartile range (IQR). Categorical variables are shown as frequencies and percentages. Data analysis and visualization was performed using GraphPad Prism (Version 10.1.1, GraphPad Software, San Diego, CA, USA).

## Results

### Patient characteristics

The study cohort comprised 18 patients. The mean age was 51 ± 17 years. Fourteen patients were male (78 %) and four were female (22 %). The median American Society of Anesthesiologists (ASA) classification was 2 (IQR 1). Arterial hypertension was the most common comorbidity (n=7, 38 %), followed by active tobacco use (n=5, 28 %) and obesity (n=3, 1 %). Detailed demographic and clinical characteristics are summarized in Table 1.

**Table 1:** Patient characteristics.

Parameter	Cohort n=18 (%)
Age, mean in years ± SD	51.3 ± 17.0
Male gender	14 (78)
ASA-Classification, median ± IQR	2 ± 1

**Risk factors**	

Arterial hypertension	7 (38)
Active tobacco use	5 (28)
Obesity (BMI≥30 kg/m^2^)	3 (17)
Diabetes	3 (17)
PAOD	3 (17)
History of thromboembolism	1 (6)

SD, standard deviation; IQR, interquartile range; BMI, body mass index; PAOD, peripheral arterial occlusive disease.

### Surgical details

All procedures were performed for traumatic soft tissue defects (n=18, 100 %). Fourteen flaps (78 %) were performed in the lower extremity and four (22 %) in the upper extremity. Lower extremity defects were localized to the foot (n=4, 28 %), the lower leg (n=5, 35 %), or both regions (n=5, 35 %). Upper extremity defects were most commonly located on the hand (n=3, 75 %).

The anterolateral thigh (ALT) flap was used most frequently (n=12, 67 %), followed by the latissimus dorsi flap (n=3, 17 %), and the parascapular and gracilis flaps. The mean operating time was 350 ± 136 min. Mean defect size was 121 ± 90 cm^2^, and mean flap size was 155 ± 52 cm^2^. Table 2 provides an overview of the flap types used.

**Table 2:** Overview over free flaps.

Free flap	Cohort n=18 (%)
ALT	12 (67)
Latissimus dorsi	3 (17)
Parascapular	2 (11)
Gracilis	1 (5)

ALT, anterolateral thigh flap.

All patients underwent osteosynthesis. Initial treatment with external fixation was performed in 10 cases (55 %). Internal plate fixation was used in 9 cases (50 %), Kirschner wires in 7 cases (39 %), while screw fixation and intramedullary nailing were less common (n=5, 28 %; n=3, 17 %). In most cases (n=9), external fixation was performed on the day of trauma; in one case, it was delayed until post-trauma day 1.

Most patients were secondary referrals (n=12, 67 %). These referrals arrived in our department on median day 25 after the trauma (IQR: 21.5 days). Secondary referrals showed prolonged treatment timelines compared to patients initially managed at our center. The median time from trauma to definitive osteosynthesis in secondary referrals was 18 days (IQR 28, range 1–33), compared to 4.5 days (IQR 17.25, range 0–22) in the primary treatment group. Similarly, soft tissue coverage was achieved later in secondary referrals, with a median of 37.5 days post-trauma (IQR 41.5, range 11–90), vs. 7.5 days (IQR 20.5, range 1–11) in those treated primarily at our institution.

The interval between final osteosynthesis and soft tissue reconstruction was also longer in secondary referrals (median 7 days, IQR 21.5, range 1–63) compared to patients initially treated in-house (median 2 days, IQR 7.25, range 1–11).

**Figure 1:**
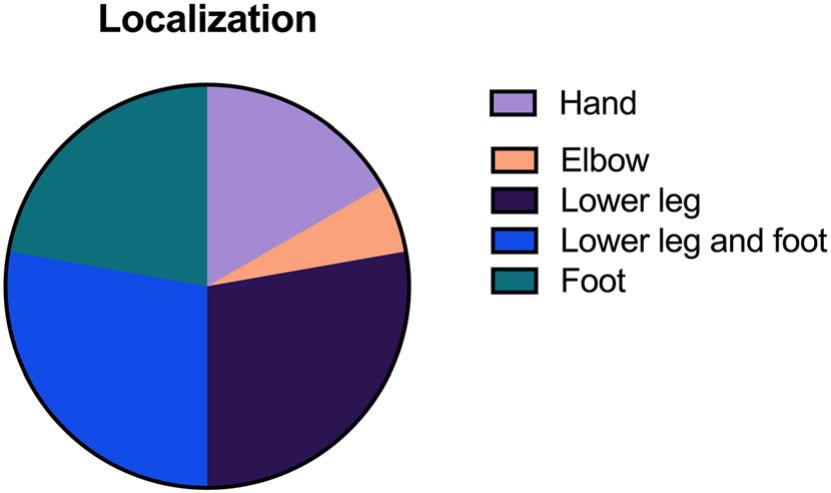
Free flap localization.

**Figure 2:**
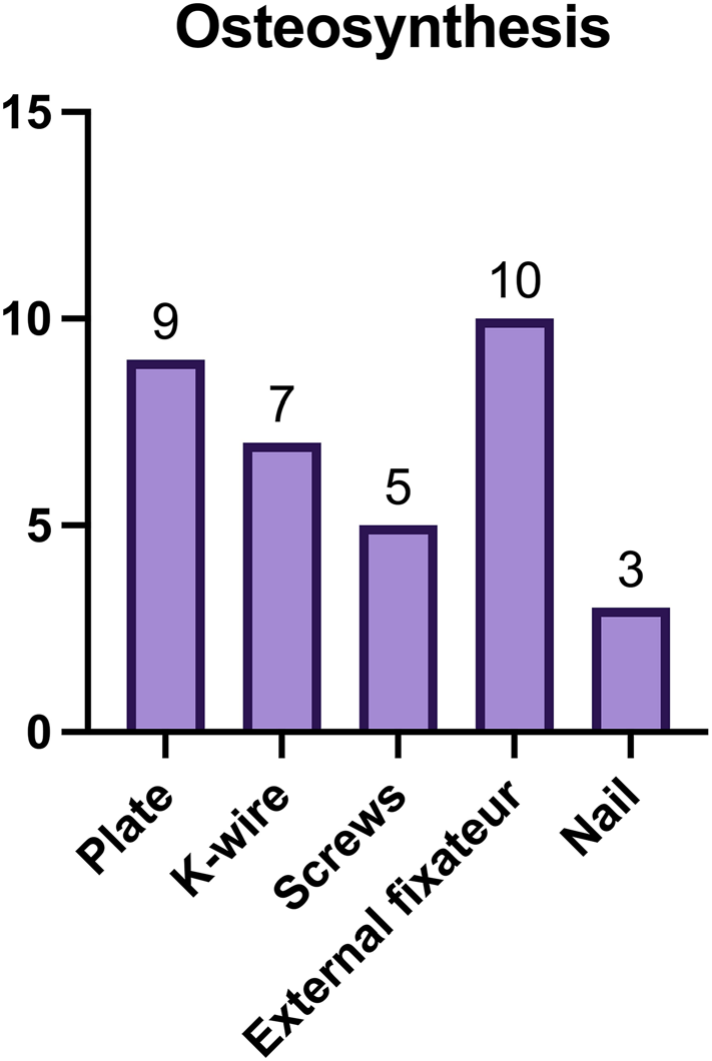
Overview over the types of osteosynthesis. Multiple entries are possible.

Figure 1 illustrates the anatomical localization of the defects, and Figure 2 provides details on osteosynthesis methods.

### Microsurgical details

**Figure 3:**
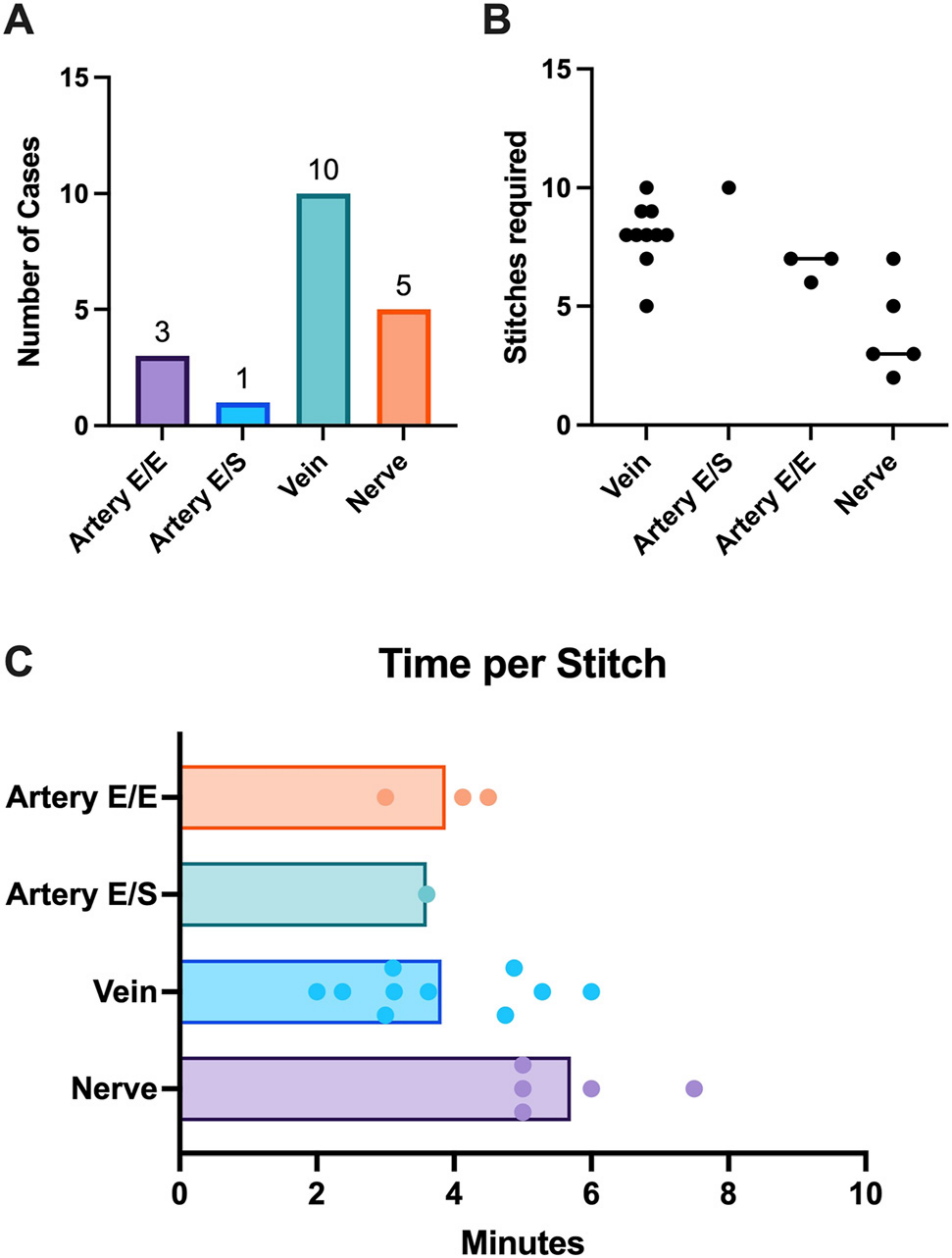
Anastomosis and coaptation characteristics. (A) Types of anastomoses and coaptations in the cohort. (B) Number of stitches required. (C) Time per stitch for each type of anastomosis/coaptation.

A total of 19 robot-assisted anastomoses and nerve coaptations were performed. Three arterial end-to-end anastomoses (n=3, 16 %) were completed with robotic assistance with a mean duration of 25.7 ± 4.2 minutes and a median of 7 sutures (IQR 0.5). A single end-to-side arterial anastomosis (n=1, 5 %) was performed in 36 minutes, requiring ten sutures. The remaining arterial anastomoses were performed conventionally in end-to-end technique in one case and end-to-side technique in 13 cases. Ten venous anastomoses (n=10, 53 %) were performed, with a mean time of 31.9 ± 11.5 minutes and a median of 8 sutures (IQR 1). The remaining venous anastomoses were performed using venous coupler devices in seven cases and hand-sewn anastomosis in one case. Five nerve coaptations (n=5, 26 %) were completed with an average duration of 22 ± 9.7 minutes and a median of 3 sutures (IQR 2). Suture materials included 8–0 and 9–0 in eight cases each (42 %), and 10–0 in three cases (15 %). No planned robot-assisted anastomoses or coaptations required intraoperative conversion to the conventional technique. Detailed data on the RAMS anastomoses and coaptations are presented in Table 3 and Figure 3.

**Table 3:** Anastomosis and coaptation details.

Anastomosis/coaptation type	Anastomoses/coaptations n=19(%)	Anastomosis/coaptation time (min, mean ± SD)	Number of stitches (median ± IQR)	Time per stitch (min, mean ± SD)
End-to-end artery	3 (16)	25.7 ± 4.2	7 ± 0.5	3.9 ± 0.8
End-to-side artery	1 (5)	36	10	3.6
Vein	10 (53)	31.9 ± 11.5	8 ± 1	4.6 ± 2.2
Nerve coaptation	5 (26)	22 ± 9.7	3 ± 2	5.7 ± 1.1

### Outcomes

Major complications were observed in one patient (5.5 %) involving an arterial thrombosis on the day of surgery. Urgent revision surgery with thrombectomy and re-anastomosis using the conventional technique successfully prevented total flap loss. However, a partial flap tip necrosis in this case remained (n=1, 5 %). No complete flap losses occurred in the entire cohort. Minor complications were documented in three patients (17 %), all involving delayed wound healing at the recipient site, which was managed conservatively through intensified local wound care.

## Discussion

This study analyzed all robot-assisted orthoplastic soft-tissue reconstructions performed at our institution between February 2023 and February 2025. A total of 18 patients underwent robot-assisted microsurgical reconstruction in an orthoplastic context. During the study period, a total of 154 robot-assisted microsurgical procedures were performed at our department. No total flap losses occurred in this cohort, although one partial flap loss was noted. Arterial thrombosis occurred in two patients (10 %) on the day of surgery. Minor complications were recorded in 17 % of cases, and a major complication in one patient (5.5 %).

Despite the limited sample size, our results are comparable to those reported in the literature. Zhang et al. reported a flap failure rate of 6.0 % and an arterial thrombosis rate of 5.0 % [20]. In earlier preliminary studies, we observed a comparable flap loss rate in a cohort of 100 robot-assisted microsurgical procedures, including 73 free flaps [7]. Nonetheless, the small sample size in the present study limits the generalizability of these findings. No intraoperative conversions to the conventional technique were required, suggesting good feasibility of RAMS in the orthoplastic setting.

Orthoplastic surgery is associated with several distinct challenges. Many patients are referred with a delay, which hinders timely intervention [21], 22]. Especially if n patients are not admitted directly to a tertiary care center, limited operating room (OR) capacity may represent a significant bottleneck. As a result, many patients – similar to the secondary referrals cohort in our study – undergo reconstruction not within days, but weeks or even months following trauma. Zhou et al. reported that only 21.5 % of the 358 orthoplastic cases in their study received soft tissue coverage within three days, and that early intervention was associated with a reduced risk of flap failure [22]. These findings have been corroborated by multiple other studies examining microsurgical outcomes in orthoplastic patient populations [23], 24]. In our institution, we therefore aim to perform soft tissue reconstruction as early as possible – at the latest on the day following internal fixation. In our study cohort, this was achieved in 4 out of 6 cases. In the remaining two cases, both involving mangled extremities, the interval was prolonged due to the need for clear demarcation of non-viable soft tissue. Furthermore, establishing effective interdisciplinary collaboration can be difficult. One potential solution that we have implemented in our institution is the implementation of regular multidisciplinary team meetings to coordinate care for complex cases [25].

In 28 % of cases (n=5), the flaps were neurotized. Rinkinen et al. reported improved sensory outcomes following free flap neurotization in foot defect reconstruction, as shown in a recent systematic review [26]. Moreover, free flap neurotization has demonstrated benefits in both head and neck as well as breast reconstruction [27], 28]. Given the growing body of evidence supporting enhanced sensory recovery, we advocate for broader implementation of flap neurotization in extremity reconstruction. Neurotization typically adds minimal operative complexity and does not increase donor or recipient site morbidity, while offering the potential for improved functional outcomes.

Robotic assistance in microsurgery offers enhanced precision, allowing surgeons to place sutures with exceptional accuracy and care, enabled by motion scaling and tremor elimination. Additionally, our preliminary preclinical study using a synthetic model indicated a steeper learning curve for robot-assisted microsurgery among novices [29]. In our experience, these advantages are most pronounced in extremely small anastomoses, such as those encountered in lymphatic surgery and supermicrosurgery. As vessel diameter increases, the number of required stitches grows, further amplifying the time difference between robotic and manual techniques. Conversely, in supermicrosurgery, where each stitch requires more time even when placed manually, the relative time disadvantage of robotic systems is reduced. This prolonged time may not translate into a clinical advantage for larger anastomoses, such as those performed in latissimus dorsi flap reconstruction. Nonetheless, robotic assistance may, in the future, enable a wider group of microsurgeons to perform highly demanding supermicrosurgical procedures, including perforator-to-perforator anastomoses. A recent meta-analysis by MacKenzie et al. identified only 25 supermicrosurgical free flap reconstructions in the head and neck region, while Lo Torto et al. reported 1,047 perforator-to-perforator flaps in a 2023 systematic review [30], 31]. These numbers indicate that perforator-to-perforator flap reconstruction has not yet become standard practice and remains limited to a small number of specialized centers worldwide. Another recognized benefit of RAMS is improved ergonomics. In a prior study, we observed significantly reduced strain on the neck and upper back when RAMS was used in combination with a digital exoscope, indicating substantial ergonomic advantages [32].

Despite its advantages, robot-assisted microsurgery continues to face notable drawbacks. Foremost among these are the high acquisition and operating costs, which may not be reimbursed depending on the healthcare system. In the case of the Symani surgical system, substantial initial capital investment is further increased by the need to purchase disposable, single-use instruments exclusively from the manufacturer. In contrast, the MUSA platform offers a potential cost advantage, as it requires replacement of only the instrument holders, while standard microsurgical instruments – already in clinical use – can be reused. In light of growing environmental awareness, the ecological burden of increased single-use components must also be considered. Future robotic platforms should thus emphasize both economic sustainability and ecological responsibility. Moreover, our data show that the time required per stitch exceeded expectations based on conventional microsurgical benchmarks. This finding may reflect both the increased interface complexity of the robotic system and the inherently slower motion resulting from motion scaling.

This study is not free of limitations. First, the retrospective nature of the study introduces inherent biases, including potential selection bias and unmeasured confounding variables. Second, the sample size of 18 cases, while sufficient for exploratory analysis, limits the power to detect subtle aspects of outcomes or complications. Third, the study was conducted at a single center, which may limit the generalizability of the results to other institutions with differing patient populations, surgical protocols, or levels of experience with robotic systems. Furthermore, the absence of a matched control group undergoing conventional orthoplastic reconstruction prevents a direct comparison of outcomes, such as complication rates, functional recovery, and patient satisfaction. Finally, granular data on the cost-effectiveness were not available, restricting the study’s ability to comprehensively evaluate the broader implications of adopting robotic technology in orthoplastic surgery.

In conclusion, we found no evidence that the incorporation of robotic assistance negatively affected outcomes in orthoplastic surgery when compared to the scientific literature. Future studies with larger cohorts and standardized outcome measures are needed to validate these findings and more definitively assess the role of robotic assistance in orthoplastic reconstruction.

## References

[1] Khan U, Boriani F, Baldini N (2013). Orthoplastics: an evolving concept for integrated surgical care of complex limb trauma and abnormality. Plast Reconstr Surg.

[2] Levin LS (1993). The reconstructive ladder. An orthoplastic approach. Orthop Clin N Am.

[3] Azoury SC, Stranix JT, Kovach SJ, Levin LS (2021). Principles of orthoplastic surgery for lower extremity reconstruction: why is this important?. J Reconstr Microsurg.

[4] Boriani F, Haq AU, Baldini T, Urso R, Granchi D, Baldini N (2017). Orthoplastic surgical collaboration is required to optimise the treatment of severe limb injuries: a multi-centre, prospective cohort study. J Plast Reconstr Aesthetic Surg.

[5] Hoyt BW, Wade SM, Harrington CJ, Potter BK, Tintle SM, Souza JM (2021). Institutional experience and orthoplastic collaboration associated with improved flap-based limb salvage outcomes. Clin Orthop Relat Res.

[6] Struebing F, Bigdeli A, Weigel J, Gazyakan E, Vollbach F, Panayi AC (2024). Robot-assisted microsurgery: lessons learned from 50 consecutive cases. Plast Reconstr Surg Global Open.

[7] Struebing F, Boecker A, Vollbach F, Weigel J, Kneser U, Bigdeli AK (2024). Robot-assisted microsurgery: a single-center experience of 100 cases. J Robotic Surg.

[8] Struebing F, Bigdeli AK, Boecker A, Weigel J, Kneser U, Gazyakan E (2024). Hands-on robotic microsurgery: robotic-assisted free flap reconstruction of the upper extremity. J Clin Med.

[9] Beier JP, Hackenberg S, Boos AM, Modabber A, Duong Dinh TA, Hölzle F (2023). First series of free flap reconstruction using a dedicated robotic system in a multidisciplinary microsurgical center. Plast Reconstr Surg Global Open.

[10] Innocenti M, Malzone G, Menichini G (2023). First-in-Human free flap tissue reconstruction using a dedicated microsurgical robotic platform. Plast Reconstr Surg.

[11] Barbon C, Grünherz L, Uyulmaz S, Giovanoli P, Lindenblatt N (2022). Exploring the learning curve of a new robotic microsurgical system for microsurgery. JPRAS Open.

[12] Aman M, Struebing F, Mayrhofer-Schmid M, Harhaus L, Kneser U, Böcker AH (2024). Bionic surgery meets bionic reconstruction – first in-human use of robotic microsurgery in targeted muscle reinnervation. Handchir Mikrochir Plast Chir.

[13] Aman M, Struebing F, Weigel J, Bigdeli AK, Gazyakan E, Kneser U (2024). Technical strategies and learning curve in robotic-assisted peripheral nerve surgery. Plast Reconstr Surg Glob Open.

[14] Schäfer B, Bahm J, Beier JP (2023). Nerve transfers using a dedicated microsurgical robotic system. Plast Reconstr Surg Glob Open.

[15] Kueckelhaus M (2024). Minimally invasive robotic-assisted perforator-to-perforator DIEP flap breast reconstruction. Plast Reconstr Surg Glob Open.

[16] Wessel KJ, Varnava C, Wiebringhaus P, Hiort M, Hirsch T, Kückelhaus M (2024). Robot-assisted microsurgery for autologous breast reconstruction – robotic breast reconstruction. Handchir Mikrochir Plast Chir.

[17] van Mulken TJM, Schols RM, Scharmga AMJ, Winkens B, Cau R, Schoenmakers FBF (2020). First-in-human robotic supermicrosurgery using a dedicated microsurgical robot for treating breast cancer-related lymphedema: a randomized pilot trial. Nat Commun.

[18] van Mulken TJM, Wolfs JAGN, Qiu SS, Scharmga AMJ, Schols RM, Spiekerman van Weezelenburg MA (2022). One-year outcomes of the first human trial on robot-assisted lymphaticovenous anastomosis for breast cancer-related lymphedema. Plast Reconstr Surg.

[19] von Reibnitz D, Weinzierl A, Barbon C, Gutschow CA, Giovanoli P, Grünherz L (2024). 100 anastomoses: a two-year single-center experience with robotic-assisted micro- and supermicrosurgery for lymphatic reconstruction. J Robot Surg.

[20] Zhang Y, Gazyakan E, Bigdeli AK, Will-Marks P, Kneser U, Hirche C (2019). Soft tissue free flap for reconstruction of upper extremities: a meta-analysis on outcome and safety. Microsurgery.

[21] Karanas YL, Nigriny J, Chang J (2008). The timing of microsurgical reconstruction in lower extremity trauma. Microsurgery.

[22] Zhou C, Al TM, Lauwers TM, Van Der Hulst RRWJ (2020). Timing of microsurgical reconstruction in lower extremity trauma: an update of the godina paradigm. Plast Reconstr Surg.

[23] Qiu E, Kurlander DE, Ghaznavi AM (2018). Godina revisited: a systematic review of traumatic lower extremity wound reconstruction timing. J Plast Surg Hand Surg..

[24] Godina M (1986). Early microsurgical reconstruction of complex trauma of the extremities. Plast Reconstr Surg.

[25] Kotsougiani-Fischer D, Fischer S, Warszawski J, Gruetzner PA, Reiter G, Hirche C (2021). Multidisciplinary team meetings for patients with complex extremity defects: a retrospective analysis of treatment recommendations and prognostic factors for non-implementation. BMC Surg.

[26] Rinkinen JR, Diamond S, Lans J, Jr CLC, Eberlin KR (2020). Neurotized free tissue transfer for foot reconstruction: a systematic review. J Reconstr Microsurg.

[27] Saha A, Mishra JK, Sahu SA, De M, Sindhuja A, Birua N (2025). Analysis of sensory recovery of neurotized free flaps in cases of head-neck reconstruction: our experience in a tertiary care hospital. Cureus.

[28] Chou J, Hyland CJ, Kaufman Goldberg T, Broyles JM (2023). Is nerve coaptation associated with improved sensation after microvascular breast reconstruction? A systematic review. Microsurgery.

[29] Struebing F, Weigel J, Gazyakan E, Siegwart LC, Holup C, Kneser U (2025). Robot-assisted microsurgery has a steeper learning curve in microsurgical novices. Life.

[30] Lo Torto F, Firmani G, Patanè L, Turriziani G, Di Rocco A, Vestri A (2024). Supermicrosurgery with perforator-to-perforator anastomoses for lower limb reconstructions – a systematic review and meta-analysis. Microsurgery.

[31] MacKenzie A, Dhoot A, Rehman U, Sarwar MS, Adebayo O, Brennan PA (2024). Use of supermicrosurgery in craniofacial and head and neck soft tissue reconstruction: a systematic review of the literature and meta-analysis. Br J Oral Maxillofac Surg.

[32] Struebing F, Gazyakan E, Bigdeli AK, Vollbach FH, Weigel J, Kneser U (2025). Implementation strategies and ergonomic factors in robot-assisted microsurgery. J Robot Surg.

